# A Pan-Cancer Study of Epidermal Growth Factor-Like Domains 6/7/8 as Therapeutic Targets in Cancer

**DOI:** 10.3389/fgene.2020.598743

**Published:** 2020-12-17

**Authors:** Shanping Shi, Ting Ma, Yang Xi

**Affiliations:** Zhejiang Provincial Key Laboratory of Pathophysiology, Diabetes Center, School of Medicine, Institute of Biochemistry and Molecular Biology, Ningbo University, Ningbo, China

**Keywords:** EGFLs, immune subtype, tumor microenvironment, survival, drug resistance

## Abstract

With highly homologous epidermal growth factor (EGF)-like (EGFL) domains, the members of the EGFL family play crucial roles in growth, invasion, and metastasis of tumors and are closely associated with the apoptosis of tumor cells and tumor angiogenesis. Furthermore, their contribution to immunoreaction and tumor microenvironment is highly known. In this study, a comprehensive analysis of EGFL6, −7, and −8 was performed on the basis of their expression profiles and their relationship with the rate of patient survival. Through a pan-cancer study, their effects were correlated with immune subtypes, tumor microenvironment, and drug resistance. Using The Cancer Genome Atlas pan-cancer data, expression profiles of EGFL6, −7, and −8, and their association with the patient survival rate and tumor microenvironment were analyzed in 33 types of cancers. The expression of the EGFL family was different in different cancer types, revealing the heterogeneity among cancers. The results showed that the expression of EGFL8 was lower than EGFL6 and EGFL7 among all cancer types, wherein EGFL7 had the highest expression. The univariate Cox proportional hazard regression model showed that EGFL6 and EGFL7 were the risk factors to predict poor prognosis of cancers. Survival analysis was then used to verify the relationship between gene expression and patient survival. Furthermore, EGFL6, EGFL7, and EGFL8 genes revealed a clear association with immune infiltrate subtypes; they were also related to the infiltration level of stromal cells and immune cells with different degrees. Moreover, they were negatively correlated with the characteristics of cancer stem cells measured by DNAs and RNAs. In addition, EGFL6, −7, and −8 were more likely to contribute to the resistance of cancer cells. Our systematic analysis of EGFL gene expression and their correlation with immune infiltration, tumor microenvironment, and prognosis of cancer patients emphasized the necessity of studying each EGFL member as a separate entity within each particular type of cancer. Simultaneously, EGFL6, −7, and −8 signals were verified as promising targets for cancer therapies, although further laboratory validation is still required.

## Introduction

Epidermal growth factor (EGF)-like (EGFL) domain gene family is named so because the protein structure of its members contains single or multiple EGFL domains. The proteins, encoded by the members of the EGFL gene family, can activate crucial signal transduction pathways such as extracellular signal-regulated kinase (ERK), nuclear factor (NF)-kappa B, mitogen-activated protein kinase (MAPK), protein kinase B, and Notch. Therefore, the gene family is widely involved in the occurrence and development of various tumors (Kang et al., 2020). The members of the EGFL family have symbolic homology with EGF and can be attributed to EGF-related proteins because they have highly homologous EGFL domains. In addition, they have common functional characteristics (Soncin et al., 2003; Parker et al., 2004; Schmidt et al., 2007). When EGFL proteins bind to their receptors, they can perform functions such as proliferation, differentiation, apoptosis, adhesion, and migration (Kang et al., 2020), indicating that the EGFL family has a crucial significance in regulating cell metastasis, tumor growth, and progression (Delfortrie et al., 2011; An et al., 2019). At present, EGFL2, EGFL3, EGFL5, EGFL6, EGFL7, EGFL8, and EGFL9 have been discovered as members of the EGFL family (Chim et al., 2013). Moreover, the most studied EGFL family members were EGFL6, EGFL7, and EGFL8. They not only have a similar structure and functional characteristics but also have a unique structure and function. They play a certain potential role in tumor occurrence, development, and prognosis because their expression is different in various tumors, indicating that they can provide new options for tumor treatment (Song et al., 2015; Kang et al., 2020). On the basis of investigations and experiments, we discussed the relationship among EGFL6, −7, and −8 and tumor development and prognosis.

Previous studies have shown that EGFL6 can activate Wnt/β-catenin and AKT/ERK signaling pathways, particularly EGFL6 plays a role in tumor angiogenesis through the ERK/AKT signaling pathway, which is associated with tumor occurrence, growth, and metastasis (Kang et al., 2020). As an example, An et al. (2019) found that EGFL6 promotes cancer cell migration, tumor angiogenesis, and tumor growth in breast cancer. In addition, EGFL7 is involved in angiogenesis through the directly or indirectly affecting the pathways mediated by Notch or vascular endothelial growth factor signaling pathways (Nichol et al., 2010). As an endogenous regulator of endothelial cell activation, EGFL7 promotes tumor progression by reducing the expression of endothelial molecules that mediate immune cell infiltration and participate in tumor immune escape mechanisms (Delfortrie et al., 2011). It has been reported that gastric cancer (GC) cells (Luo et al., 2014), liver cancer (HCC) cells (Shen et al., 2016), and pituitary adenocarcinoma cells (Wang et al., 2017) have considerably improved migration and invasion capabilities because of the overexpression of EGFL7. However, compared with normal tissues, the expression level of EGFL8 is significantly reduced in colorectal cancer (CRC) tissues (Wu et al., 2011b) and gastric cancer tissues (Wu et al., 2011a). Moreover, a link has been established between the downregulation of EGFL8 and CRC (Wu et al., 2011b) and GC (Song et al., 2015) metastasis and poor prognosis. In conclusion, EGFL6, 7, and 8 are more likely to offer a breakthrough for cancer therapy. In fact, nobody until the date has conducted a systematic study of these EGFL family genes in various tumors. Each gene has only been analyzed in several cancer types, and the majority of studies have been based on the use of cell lines and/or animal models. However, this study performed a systematic pan-cancer analysis of EGFL6, 7, and 8, comprehensively describing their characteristics and their importance in tumor research.

In this study, The Cancer Genome Atlas (TCGA)-pan-cancer data were used to analyze the expression patterns of EGFL6, EGFL7, and EGFL8 of the EGFLs family and their relationship with the overall survival rate in 33 primary tumors of patients and associate their expression with tumor microenvironment and pharmacological activity. In different tumors, these three genes display inconsistent upregulation or downregulation. The association between gene expression and overall survival depends on the subtype queried, and the type of cancer tested. In addition, EGFL6, EGFL7, and EGFL8 were found to be related to immune subtypes and tumor microenvironment. In addition, the degree of association differs in each family member and tumor type. Our results concretely establish that these three genes were linked with the tumor stem cell-like characteristics and resistance to chemotherapeutic drugs. This study emphasized the distinct difference of the EGFL members, which exists among various tumor types and the necessity of studying each EGFL member as a separate entity.

## Materials and Methods

### The Cancer Genome Atlas Pan-Cancer Data

TCGA pan-cancer data included RNA-Seq (RNA SeqV2 RSEM), clinical data, stemness scores, which were on the basis of mRNA (RNAs) and DNA-methylation (DNAs), and immune subtypes which could be downloaded from Xena Browser^1^. In TCGA, the tumor samples are surgically resected and taken from primary tumors that have never received neoadjuvant therapy. In the analysis of intertumor/pan-tumor, gene expression should be standardized to TATA-binding protein (TBP). The TCGA pan-cancer data consist of 33 types of cancers: adrenocortical carcinoma (ACC), bladder urothelial carcinoma (BLCA), Breast invasive carcinoma (BRCA), cholangiocarcinoma (CHOL), Cervical squamous cell carcinoma and endocervical adenocarcinoma (CESC), colon adenocarcinoma (COAD), lymphoid neoplasm diffuse large B-cell lymphoma (DLBC), esophageal carcinoma (ESCA), glioblastoma multiforme (GBM), head and neck squamous cell carcinoma (HNSC), kidney chromophobe (KICH), kidney renal clear cell carcinoma (KIRC), kidney renal papillary cell carcinoma (KIRP), acute myeloid leukemia (LAML), brain lower grade glioma (LGG), liver hepatocellular carcinoma (LIHC), lung adenocarcinoma (LUAD), lung squamous cell carcinoma (LUSC), mesothelioma (MESO), ovarian serous cystadenocarcinoma (OV), pancreatic adenocarcinoma (PAAD), pheochromocytoma and paraganglioma (PCPG), prostate adenocarcinoma (PRAD), rectum adenocarcinoma (READ), sarcoma (SARC), skin cutaneous melanoma (SKCM), stomach adenocarcinoma (STAD), stomach and esophageal carcinoma (STES), testicular germ cell tumors (TGCT), thyroid carcinoma (THCA), thymoma (THYM), uterine corpus endometrial carcinoma (UCEC), uterine carcinosarcoma (UCS), uveal melanoma (UVM). A total of 11,057 tissues samples were useful for the study, and there were 521 colorectal cancer samples, including 41 adjacent samples; however, the quality of samples varied in cancer types, such as there were more than 1,000 samples for breast cancer, but only 45 samples for cholangio carcinoma. Among them, 15 cancer types had none or less than five associated normal tissue samples; therefore, the remaining 18 cancer types were used to investigate whether there was altered gene expression in tumors compared with adjacent normal tissues using linear mixed-effects models. In fact, only 18 cancer types were used to analyze whether the gene expression in tumors had changed compared with the adjacent normal tissues using the linear mixed-effects model. For investigating the association between gene expression (as persisted variable) of each member of the EGFL family and overall survival rate of patients with cancer, apart from THYM, which had no patient survival data, all patient tumor samples were available for survival analysis.

### Tumor Microenvironment Analysis

In various tumors, the infiltration levels of immune cells and stromal cells were investigated using the ESTIMATE immune and stromal scores (Yoshihara et al., 2013). The tumor purity was described using the ESTIMATE score from this program. The explanation of gene expression profiles by searching the TCGA expression data^2^ gave birth to this analysis (Yoshihara et al., 2013). Spearman’s correlation was used to test the relationship between the expression of EGFL members and the scores. To measure immune infiltration in the tumor environment, six immune subtypes were defined (Thorsson et al., 2018). By using analysis of variance models, immune subtype obtained from TCGA pan-cancer data was used to determine the relationship between the expression of EGFL6, EGFL7, and EGFL8 and immune infiltrate types in the tumor microenvironment. In TCGA tumor samples, tumor stemness characteristics obtained from epigenetic and transcriptome were used to measure stem cell-like characteristics of tumor cells (Malta et al., 2018). The association between cancer stemness and the expression of EGFL6, EGFL7, and EGFL8 was determined using Spearman’s correlation test.

### National Cancer Institute-60 Analysis

The National Cancer Institute (NCI)-60 database was accessed using the CellMiner interface, which includes data on 60 different cancer cell lines from nine different tumors^3^. mRNA expression levels of EGFL members and z scores of 59 cell lines were used to obtain the cell sensitivity data (GI50). In addition, the association between gene expression and drug sensitivity was studied using Pearson’s correlation coefficient. The drugs involved in the correlation analysis included the drugs of 262 drug reactions approved by the Food and Drug Administration or the drugs on clinical trials.

### Statistical Analyses

Only 18 tumors showed a higher level of gene expression compared to five related adjacent normal samples. Therefore, only these 18 tumors were included for the comparison of the degree of gene expression between the normal and tumor tissues using the linear mixed-effects models. Boxplots can clearly reveal the difference in gene expression among cancer types. Assessing the association between gene expression and patient’s overall survival depended on the univariate or multivariate Cox proportional hazard regression models or log-rank tests. The association between gene expression and stemness scores, stromal score, immune score, estimate score, and drug sensitivity was determined using Spearman’s or Pearson’s correlation. The correlation between gene expression and patient clinical features and immune compositions was verified using linear regressions. All of the tests were performed using SAS9.4 (SAS Institute Inc., NC). Under appropriate conditions, plots were prepared by R (RCore Team) using the packages of ggplot2, pheatmap, corrplot, or survminer. Apart from the survival study, for controlling the rate of familywise error at α = 0.05, the number of false positives method was used to adjust a lot of comparisons for all of the tests. Specifically, on the basis of assuming one false positive in all the tests of each study, we set the critical value of *P* for significance. For instance, because of 54 tests (3 genes × 18 cancer types) in this study, we considered α = 1/(3 × 18) = 0.05 as the cutoff to compare the gene expression of EGFL members between tumor tissues and adjacent normal tissues. In the survival study, for interpreting the data in line with the results shown in the forest plots, α = 0.05 was considered the cutoff without multiple comparisons. However, gene expression between tumor tissues and normal tissues was compared using the same method that controlled multiple comparisons to establish an obvious correlation between gene expression and overall survival, i.e., *P* < 1/(3 genes × 32 cancer types) = 0.05 was significant.

## Results

### EGFL Domains Gene Expression in Pan-Cancer

**FIGURE 1:**
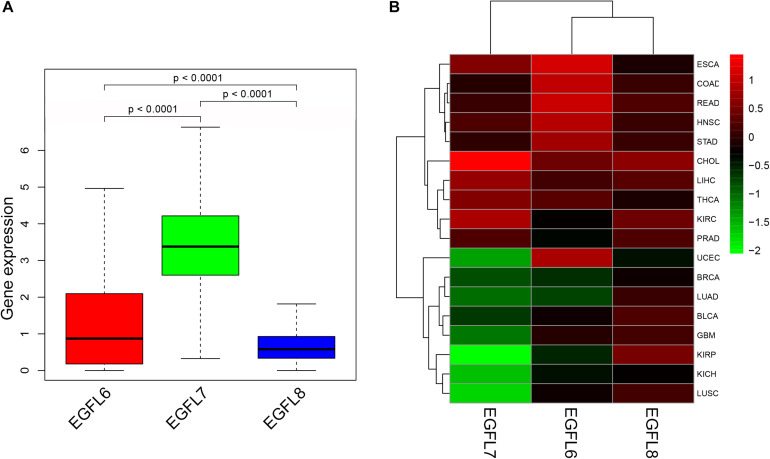
Expression levels of EGFL6/7/8 in cancerous and adjacent normal tissues. **(A)** Boxplot showing the distribution of EGFL6/7/8 gene expression across all 33 cancer types. **(B)** Heatmap showing the difference of EGFL6/7/8 gene expression in comparison to the primary tumor to the adjacent normal tissues based on log2 (fold change) for 18 cancer types that have more than five adjacent normal samples.

In order to understand the intrinsic expression pattern of EGFL genes, the expression degrees of EGFL6, 7, and 8 were determined in 33 types of cancers, which are available in TCGA pan-cancer data (Supplementary Table 1). Our study showed a striking inter-tumor heterogeneity in the expression levels of the corresponding genes for three EGFL members (Figure 1A). An apparent heterogeneity was observed in the gene expression of each EGFL gene among different tumor types. In a few tumor types, the gene was expressed at a high level, whereas in other tumors, the expression was negligible (Supplementary Figure 1). For instance, few tumors having extremely low levels of EGFL6 were CHOL, GBM, KICH, KIRC, KIRP, LIHC, PRAD, and THCA, whereas those having a high level of EGFL6 expression were BLCA, ESCA, HNSC, LUAD, LUSC, and UCEC. The largest inter-tumor heterogeneity was significantly observed in the expression levels of EGFL6 (Figure 1A and Supplementary Figure 1). The expression levels of EGFL8 were relatively lower among all cancer types than that of EGFL6 and EGFL7, with EGFL7 having the highest level of expression (Figure 1A). Significant differences were observed in the expression of EGFL gene family among different types of tumors, as well as among different members of EGFL family within each tumor type, demonstrating the need to study each gene member as a separate entity. In tumorigenesis, one typical functional feature of these genes was their expression dysregulation in tumors, and increasingly, evidence supported the conclusion that the expression of the EGFL family members changes in different tumors (Wu et al., 2011a; Nichol and Stuhlmann, 2012; Song et al., 2015). However, the majority of the evidence has been provided by the studies on cell lines or animal models. Our study analyzed the expression levels of EGFL6, EGFL7, and EGFL8 in 18 primary tumors that had more than five adjacent normal samples (Figure 1B). All EGFL members clearly displayed different expression in various types of cancers. Whereas EGFL6 and EGFL8 were mainly upregulated in the tested tumors, and EGFL7 was upregulated in approximately one-half of the tested tumors and downregulated in the other half.

### Association of EGFL Domains Gene Expression With Patient Overall Survival and Disease-Free Survival

**FIGURE 2:**
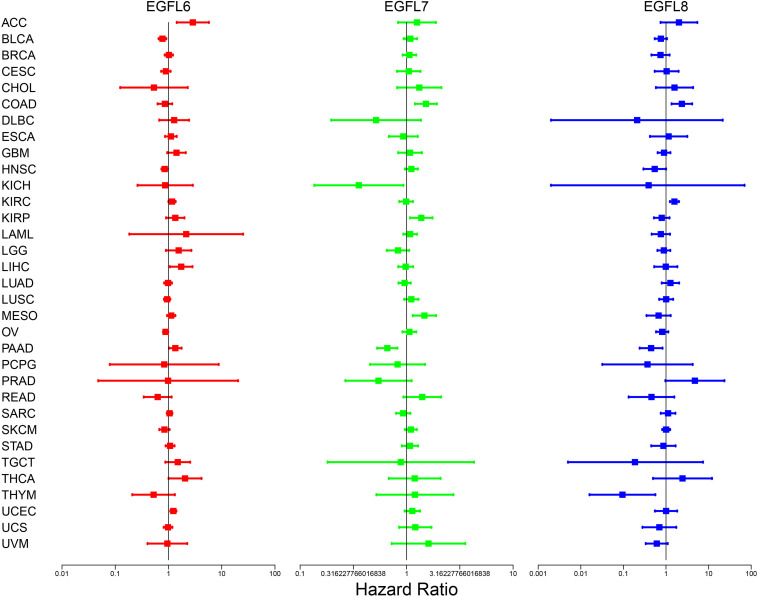
Association of EGFL6/7/8 gene expression with patient’s overall survival for different cancer types. The forest plots with the hazard ratios and 95% confidence intervals for overall survival for different cancer types showing the survival advantage and disadvantage with the increased gene expression of the EGFL6/7/8 family. The univariate Cox proportional hazard regression models were used for the association tests.

To clearly determine the roles that each EGFL family member plays in different tumors, we used 33 types of primary cancers to evaluate the association between EGFL gene expression and patient overall survival. The analysis was based on the univariate Cox proportional hazard regression models; we alleged obvious correlation with *P* < 0.05, and adjusting for multiple comparisons was not required to be in line with the forest plots, as shown in Figure 2. The change in the expression of EGFL members was typically associated with the patient’s overall survival; however, the correlated direction differed in the EGFL member queried, and the cancer type tested as shown in Figure 2. Specifically, the high expression of EGFL6 and EGFL7 was mainly associated with survival disadvantage. EGFL6 predicted the poor prognosis in patients with ACC, KIRC, LIHC, PAAD, THCA, and UCEC (*P* < 0.05), whereas EGFL7 predicted poor prognosis in the patients with COAD, KIRP, and MESO (*P* < 0.05). The results indicated that they can be used as an independent risk factor for these cancers. The association between EGFL8 and survival advantage or disadvantage differed in the cancer types. Specifically, EGFL8 predicted poor prognosis for patients with COAD and KIRC, whereas it predicted a survival advantage for patients with PAAD and THYM (*P* < 0.05) (Supplementary Table 2). We then performed a survival analysis to determine the association between EGFL6, −7, and −8 and cancers. Notably, the overexpression of EGFL6 and EGFL8 was associated with better survival in patients with READ and HNSC and poor survival in patients with KIRC. EGFL6 and EGFL7 were both related to poor survival in patients with KIRP (*P* < 0.05) (Supplementary Figure 2).

Furthermore, we retrieved the clinical information, including disease-free survival and status for all patients from the TCGA database. The univariate analysis of the Log-rank test was done for the three genes, independently. KM curves were drawn to show the diverse disease-free prognosis of patients with diverse expression levels of the three genes. We also applied a multivariate survival analysis method of CoxPh regression to assess the interaction between the three genes. A forest graph was shown using the HR estimated by CoxPh. It suggests that EGFL6, EGFL7, and EGFL8 were significantly related to disease-free survival, and the prognosis was better when EGFL6 and EGFL8 were highly expressed, and the prognosis was better when EGFL7 is low. The above figures are shown in Supplementary Figure 3.

### EGFL Domains Genes Associated With Immune Response and Tumor Microenvironment in Cancer

**FIGURE 3:**
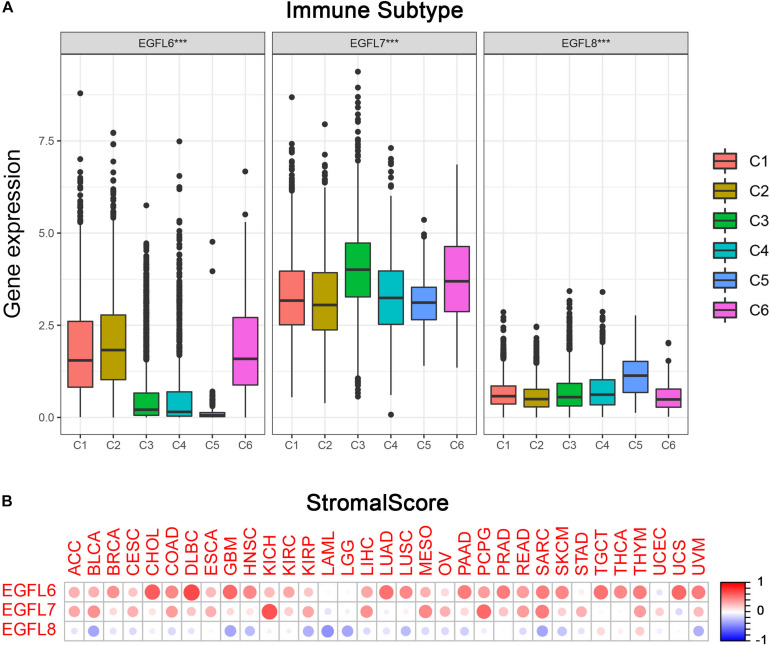
Association of EGFL6/7/8 gene expression with tumor microenvironment factors. **(A)** Association of EGFL6/7/8 gene expression with immune infiltrate subtypes across all the cancer types (*P* < 0.05) tested with ANOVA. **(B)** Correlation matrix plots showing the association between EGFL6/7/8 gene expression and stromal scores of 33 different cancer types based on ESTIMATE algorithm. Spearman’s correlation was used for testing. The size of the dots stands for the absolute value of the correlation coefficients. The bigger the size is, the higher the correlation would be (higher absolute correlation coefficient). This also applies to Figures 4A,B, as well as Supplementary Figures S4A,B.

Previous studies have demonstrated that EGFL family members have several potential functions in the immune reaction; for example, EGFL7 promotes immune escape mechanism by decreasing the immune cell infiltration (Delfortrie et al., 2011; Pinte and Soncin, 2012). To understand the association between EGFL6, 7, and 8 and immune components, we examined the relation between EGFL and immune infiltrates in tumors. Six types of immune infiltrates have been identified in human tumors which can promote or inhibit tumors (Tamborero et al., 2018), which are as follows: C1 (wound healing), C2 (interferon (IFN)-r dominant), C3 (inflammatory), C4 (lymphocyte depleted), C5 (immunologically quiet), and C6 [tumor growth factor β (TGF β) dominant]. On the basis of the rate of overall survival among all cancer types, patients with C3 and C5 immune subtypes had better survival than those with other subtypes (*P* < 0.0001), particularly the patients with types C4 and C6 had the lowest survival, the pairwise p values for all the immune subtypes are already shown in Supplementary Figure 4 (Tamborero et al., 2018). We investigated the immune infiltration in the TCGA pan-cancer data and associated them with the expression levels of EGFL6, 7, and 8 (Figure 3A).

As a high level of EGFL6 was associated with types 1, 2, and 6 infiltration (C1, C2, and C6), EGFL6 was concluded to be a tumor promoter because patients with these types had worse survival with a high proliferation rate and rich TGF β (Figure 3A and Supplementary Figure 4). In contrast, compared with other infiltrate types, the correlation between the high expression of EGFL8 and C5 was observed, suggesting that EGFL8 was associated with a favorable immune component, indicating that EGFL8 may mainly act as a tumor suppressor. EGFL7 is crucial and highly expressed in all six types of immune infiltrates. Although C3 has the highest expression, its relationship with prognosis needs to be further analyzed.

More interestingly, there was an extensive scope in the degree of association between members of the EGFL family and stromal score for various cancer types (Figure 3B). EGFL6 had the highest correlation with the stromal score among all cancer types (*r* = 0.71, *P* < 0.001), following with EGFL7 (*r* = 0.65, *P* < 0.001) and EGFL8 (*r* = 0.19, *P* < 0.001). In CHOL, DLBC, GBM, LUAD, PAAD, PRAD, SARC, TGCT, THYM, UCS, and UVM, a clear positive correlation between EGFL6 and the stromal score was observed. In addition, EGFL7 was positively associated with the stromal score in KICH, MESO, PCPG, and SARC. The results showed that EGFL6 and EGFL7 had a worse prognosis in these cancers (Wang et al., 2019). However, EGFL8 clearly showed a negative correlation with the stromal score in BLCA, GBM, LAML, LGG, and SARC (*P* < 0.0001). In addition, we assessed the correlation of EGFL members with immune and ESTIMATE scores that measure the level of immune cell infiltrates and tumor purity using the ESTIMATE program (Yoshihara et al., 2013; Becht et al., 2016), and we obtained parallel results to the test of the stromal score (Supplementary Figure 5).

Thus, EGFL6 and EGFL7 had a similar performance in tumor immunity and micro-environment. Considering an example of CRC, we searched for genes with a high correlation coefficient (Pearson’s correlation coefficient >0.5) and made an intersection. Finally, 74 genes were selected for the next functional analysis. Biological process (BP) and Kyoto Encyclopedia of Genes and Genomes (KEGG) analyses were performed through the DAVID website^4^. Detailed information is summarized in Table 1, among which five BPs were observed, which were as follows: regulation of angiogenesis and vasculature development, vasculogenesis, endothelium development, positive regulation of angiogenesis, and positive regulation of vasculature development. In KEGG analysis, cell adhesion molecules, MAPK signaling pathway, hematopoietic cell lineage, NF-kappa B signaling pathway, and *Salmonella* infection were involved. These genes were associated with tumor angiogenesis. “MAPK signaling pathway” suggests that they are involved in the process of cell differentiation and growth. NF-kappa B signaling plays a crucial role in inflammation and immune response, which may be an important signal pathway for EGFL6 and EGFL7 to interfere with tumor immunity and tumor stromal cell infiltration.

**TABLE 1:** BP and KEGG analysis.

	**Counts**	***P-*value**
**Enriched BP term**		
Regulation of angiogenesis	14	3.96E-11
Regulation of vasculature development	14	1.12E-10
Vasculogenesis	8	3.64E-10
Endothelium development	9	5.74E-10
Positive regulation of angiogenesis	9	3.03E-08
Positive regulation of vasculature development	9	7.68E-08
**Enriched KEGG term**		
Cell adhesion molecules	4	0.002
MAPK signaling pathway	5	0.004
Hematopoietic cell lineage	3	0.005
NF-kappa B signaling pathway	3	0.006
Salmonella infection	4	0.007

### EGFL Domains Genes Associated With Tumor Stemness and Cancer Cell Sensitivity to Chemotherapy

**FIGURE 4:**
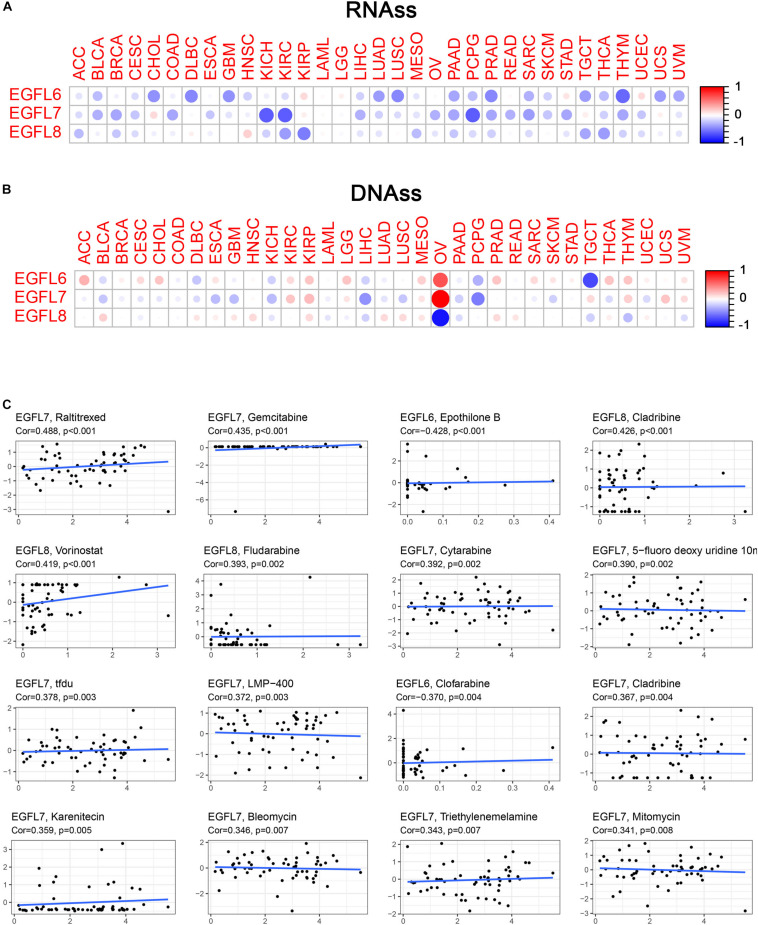
Association of EGFL6/7/8 gene expression with tumor stemness and drug sensitivity. **(A,B)** Correlation matrix between EGFL6/7/8 gene expression and cancer stemness scores RNAss **(A)** and DNAss **(B)**, respectively, based on Spearman’s correlation tests. **(C)** Scatter plots showing the association between EGFL6/7/8 gene expression and drug sensitivity (*Z*-score from CellMiner interface) tested by Pearson’s Correlation using NCI-60 cell line data.

With the progression of cancer, differentiated phenotypes of tumor cells disappeared one by one, and the cells obtained progenitor and stem cell-like characteristics. The studies have shown that in tumor-initiating stem cells, the expression of EGFL members had an increasing trend and played pivotal roles in tumor resistance. For example, in ovarian cancer, overexpression of EGFL6 was associated with drug resistance (Januchowski et al., 2014). To measure the tumor stemness, RNA and DNA stemness scores were used on the basis of mRNA expression (RNAs) and DNA methylation pattern (DNAs), respectively (Malta et al., 2018). In this study, the association between EGFL genes and tumor stemness measured by RNAs and DNAs was investigated. The degree of association with RNAs and DNAs in various cancer types was different, which was a characteristic of the EGFL family (Figures 4A,B). Notably, EGFL8 had a negative association with RNAs and DNAs (*P* < 0.001), and EGFL8 had the strongest correlation with DNAs (*r* = -0.89) in all cancer types. EGFL6 and EGFL7 had a significantly clear negative correlation with DNAs and RNAs in PCPG; however, in OV, the genes were positively correlated with DNAs and negatively with RNAs. These inconsistent outcomes indicated that RNAs and DNAs can be used to identify diverse populations of cancer cells based on different characteristics or degrees of stemness in various cancer types (Malta et al., 2018). As EGFL genes are constantly associated with stem cell-like characteristics, we analyzed the expression of EGFL in 60 human cancer cell lines and examined the correlation between their expression levels in NCI-60 cell lines (Supplementary Table 3). *Z*-score was used to measure drug sensitivity; the scores showed the cell sensitivity to the drug, and the sensitivity increased with an increase in the score. In addition, the increase in the expression of EGFL members, particularly EGFL7 and EGFL8, was associated with an increase in drug resistance of distinct cell lines to multiple chemotherapy drugs (*r* > 0.3 and *P* < 0.01) (Figure 4C). Notably, EGFL6, 7, and 8 were associated with the sensitivity of several drugs. In addition, the roles of different genes were likely to have an opposite association for the same drug. For instance, for cladribine, EGFL8 was associated with the increase in cell sensitivity, whereas EGFL6 was related to the increase in cell resistance.

### EGFL Domain Gene Family in Colorectal Cancer

**FIGURE 5:**
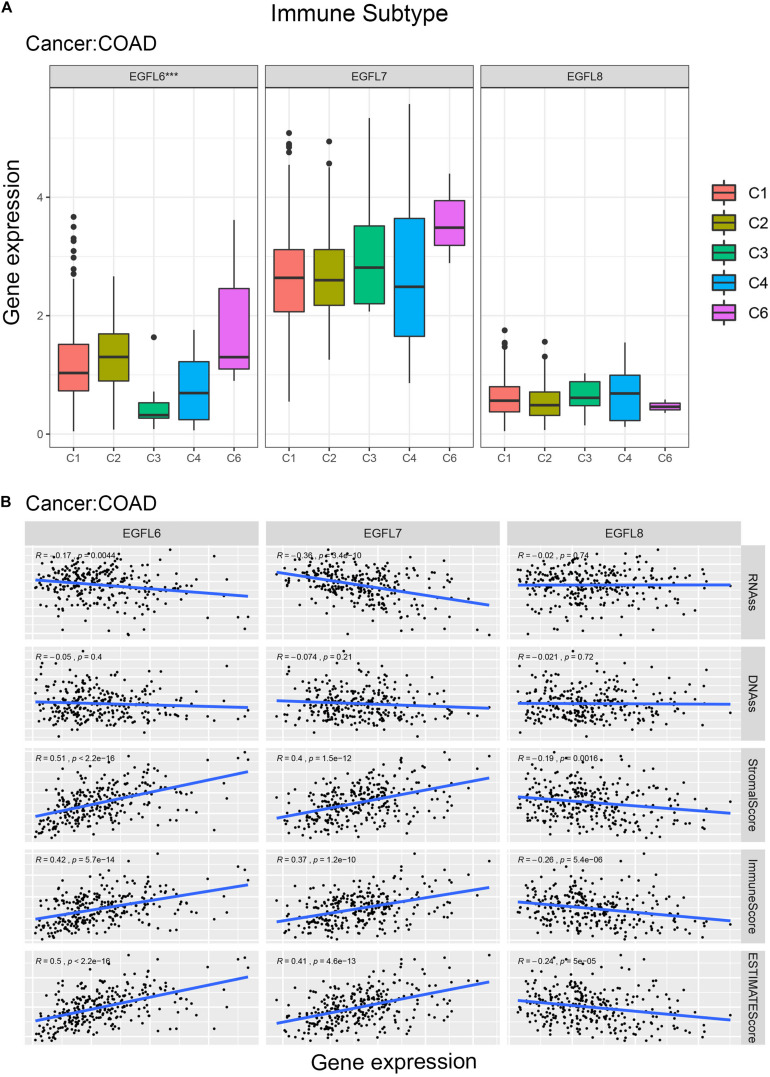
EGFL6/7/8 gene expression in colorectal cancer. **(A)** Association of EGFL6/7/8 gene expression with immune infiltrate subtypes in colorectal cancer tested with ANOVA (*P* < 0.05). **(B)** Correlation matrixes between EGFL6/7/8 gene expression and RNAss, DNAss, stromal score, immune score, and Estimate Score. Spearman’s correlation tests were used for testing.

EGFL6, 7, and 8 have been partially studied in CRC (Song et al., 2015; Hong et al., 2018; Zhang et al., 2019). In this study, TCGA CRC data were used to conduct a comprehensive study of EGFL genes in one of the largest CRC patient cohort publicly available. Compared with the adjacent normal tissues, the expression of EGFL6 was clearly different in CRC (*P* < 0.01) (Supplementary Figure 6). The pattern of the association between the gene expression of EGFL6 and eight and immune subtypes in CRC was similar to that observed in the use of all 33 TCGA tumors in all cancer types; EGFL6 was significantly associated with immune infiltrate types (*P* < 0.001) (Figure 5A). In the tumor microenvironment, particularly in CRC, stromal cells can be considered a large compartment. Our further study investigated the correlation between the expression of EGFL and stromal score. EGFL6 and EGFL7 had a positive correlation with stromal scores (*r* = 0.51, 0.40, respectively, and *P* < 0.001) in CRC, suggesting that tissue stroma in CRC may express them (Figure 5B). In contrast, EGFL8 was negatively correlated with stromal scores (*r* = -0.19 and *P* = 0.0016). In addition, they were associated with an immune score, and the score can measure the existence of infiltrating immune cells (*P* < 0.001) and tumor purity (ESTIMATE score) (*P* < 0.001) (Figure 5B). EGFL6 and EGFL7 showed a negative correlation with RNA stemness score (*r* = -0.17, −0.36, respectively, *P* < 0.001), and all of them showed a smaller degree of association with DNAs (Figure 5B).

## Discussion

In recent years, several studies have shown that EGFL6, EGFL7, and EGFL8 play a crucial role in the process of tumor growth, invasion, and distant metastasis. Moreover, they are involved in the apoptosis of tumor cells and tumor angiogenesis. In brief, these three members of the EGFL gene family stimulate or inhibit tumor growth by affecting tumor cells and their microenvironment. In addition, they have been widely investigated as targets for tumor therapy.

Our study provided the first systemic pan-cancer analysis of the genes. In addition, large differences were observed in the expression levels of EGFL6, EGFL7, and EGFL8 among different tumor types and compared with normal tissues (*P* < 0.05). This suggested that they are likely to be biological markers of the tumor. For instance, Wu et al. (2009) found that EGFL7 is highly expressed in liver cancer tissues, which are linked to liver cancer metastasis and may be a metastasis and prognostic marker of liver cancer. We then further assessed the association between the expression of EGFL members and patient’s overall survival in 33 cancer types and found that the results varied in different cancer types, but EGFL6 and EGFL7 were mainly associated with poor prognosis. Therefore, their expression is a risk factor for multiple tumor progression. For example, their high expression is associated with the poor overall survival rate of the KIRP. Moreover, in this study, these three EGFL family members were significantly associated with immune infiltrate subtypes in the tumor microenvironment, where EGFL6 and EGFL7 were related to more aggressive subtypes of immune infiltrates, i.e., C1, C2, and C6, and were rich in IFN-r infiltration, indicating a correlation with promoting tumor progression. However, EGFL8 showed the opposite result; it had noticeably higher expression in immune infiltrate C5 than in others, indicating that it was associated with good immune components, implying that this gene may mainly inhibit tumors. These findings have been partially verified in previous studies; for instance, the downregulation of EGFL8 expression in gastric cancer was clearly associated with distant metastasis and invasion of lymphoid nodes (Wu et al., 2011a; Song et al., 2015), whereas the migration and invasion ability of gastric cancer cells was enhanced because of the overexpression of EGFL7 in gastric cancer and the epithelial-mesenchymal transition (EMT) process is also promoted (Luo et al., 2014). The poor performance of EGFL6 in various cancers, including nasopharyngeal carcinoma (Zhu et al., 2018), lung adenocarcinoma (Chang et al., 2018), ovarian cancer (Bai et al., 2016), etc. may be associated with its remarkable immunosuppressive effect in the tumor immune microenvironment. This should be further investigated and verified. On the basis of the ESTIMATE algorithm, EGLF6, EGFL7, and EGFL8 were also related to the different degrees of tumor-stromal cell and immune cell infiltrates. The findings showed that they can function as proinflammatory and immune modulators (Delfortrie et al., 2011; An et al., 2019). Finally, we analyzed the correlation between the genes and tumor stemness score and the drug sensitivity score. However, all these results need to be further verified in the laboratory, and EGLF6, EGFL7, and EGFL8 are likely to be promising therapeutic targets.

In addition, a correlation analysis was performed on the expression levels of the three genes in different high-incidence tumors; but the results revealed that only two of the genes were highly correlated with individual types of tumors. However, this does not indicate that they have no common biological functions; for example, they are jointly involved in tumor angiogenesis (Wu et al., 2011a; Nichol and Stuhlmann, 2012; Noh et al., 2017). Therefore, EGLF6, EGFL7, and EGFL8 had a significantly negative association with tumor stem cell-like features measured by DNAs and RNAs; however, their negative correlation in RNAs was not prominent and only manifested in individual tumors. In contrast, they had a positive correlation with stem cell-like characteristics measured using DNAs, showing that they may play roles in tumor-initiating cells and be associated with cancer cell resistance to drug treatment. Although this study only showed a significant difference in the expression of EGFL6 in CRC (Supplementary Figure 6), previous studies have shown that EGFL7 was highly expressed (Fan et al., 2013), and EGFL8 was significantly lower (Wu et al., 2011b) in CRC. The difference can be attributed to the fact that this study considered EGFL7 and EGFL8 expression at the mRNA level, whereas the expression of EGFL7 and EGFL8 was measured at a protein level in tissue microarrays in the previous study.

In this pan-cancer study, a comprehensive and systematic description of the features of these three genes was provided, and the need to study their functions in the cancer type and the dependency of immune subtype was highlighted. Overall, our results proved that EGFL6 and EGFL7 more frequently promoted tumorigenesis and were associated with poor prognosis, and EGFL8 more frequently inhibited tumorigenesis and was typically associated with better prognosis. However, the presumptive tumor promoter or tumor suppressor effect of EGFL members was inconsistent among other family members within a specific cancer type. In conclusion, our study showed their roles in tumorigenesis, particularly in immune reaction, tumor microenvironment, and drug resistance, which is crucial to develop personalized medicine for cancer treatment.

## Data Availability Statement

The original contributions presented in the study are included in the article/Supplementary Material, further inquiries can be directed to the corresponding author.

## Author Contributions

SS and YX helped conceive and design and contributed to data analysis. TM contributed to data analysis and interpretation. All authors read and approved the final manuscript.

## Conflict of Interest

The authors declare that the research was conducted in the absence of any commercial or financial relationships that could be construed as a potential conflict of interest.

## Footnotes

1 https://xenabrowser.net/datapages/

2 http://bioinformatics.mdanderson.org/estimate/

3 https://discover.nci.nih.gov/cellminer/

4 https://david.ncifcrf.gov
