# Supplementary material for: A Pan-Cancer Study of Epidermal Growth Factor-Like Domains 6/7/8 as Therapeutic Targets in Cancer
Source: Front Genet. 2020 Dec 17;11:598743. doi: 10.3389/fgene.2020.598743 (PMC7773905; doi:10.3389/fgene.2020.598743)
Supplement: Supplementary file 1 [file Data_Sheet_1.zip › supplementary figure.pptx]

## Slide 1
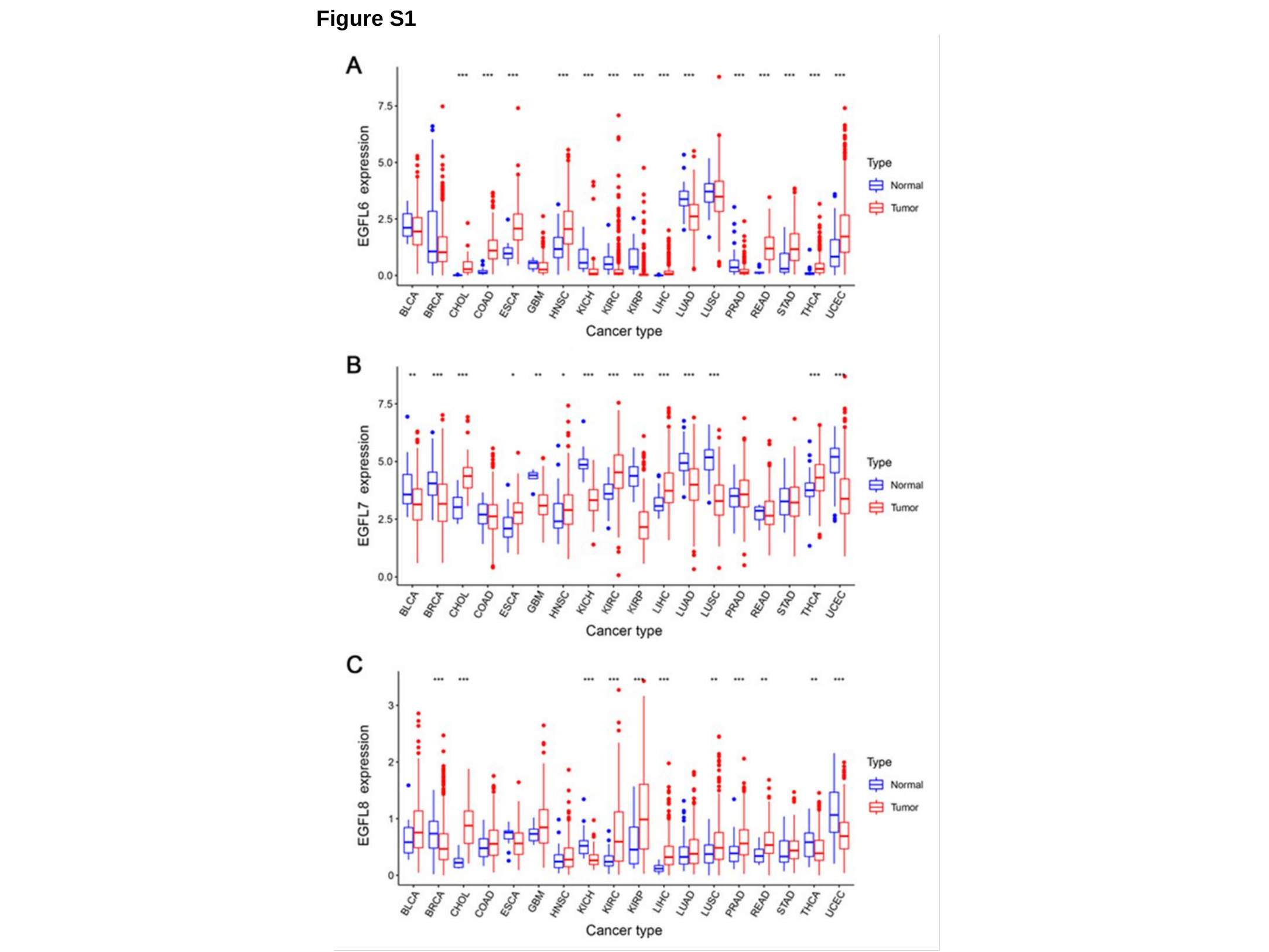

Figure S1

## Slide 2
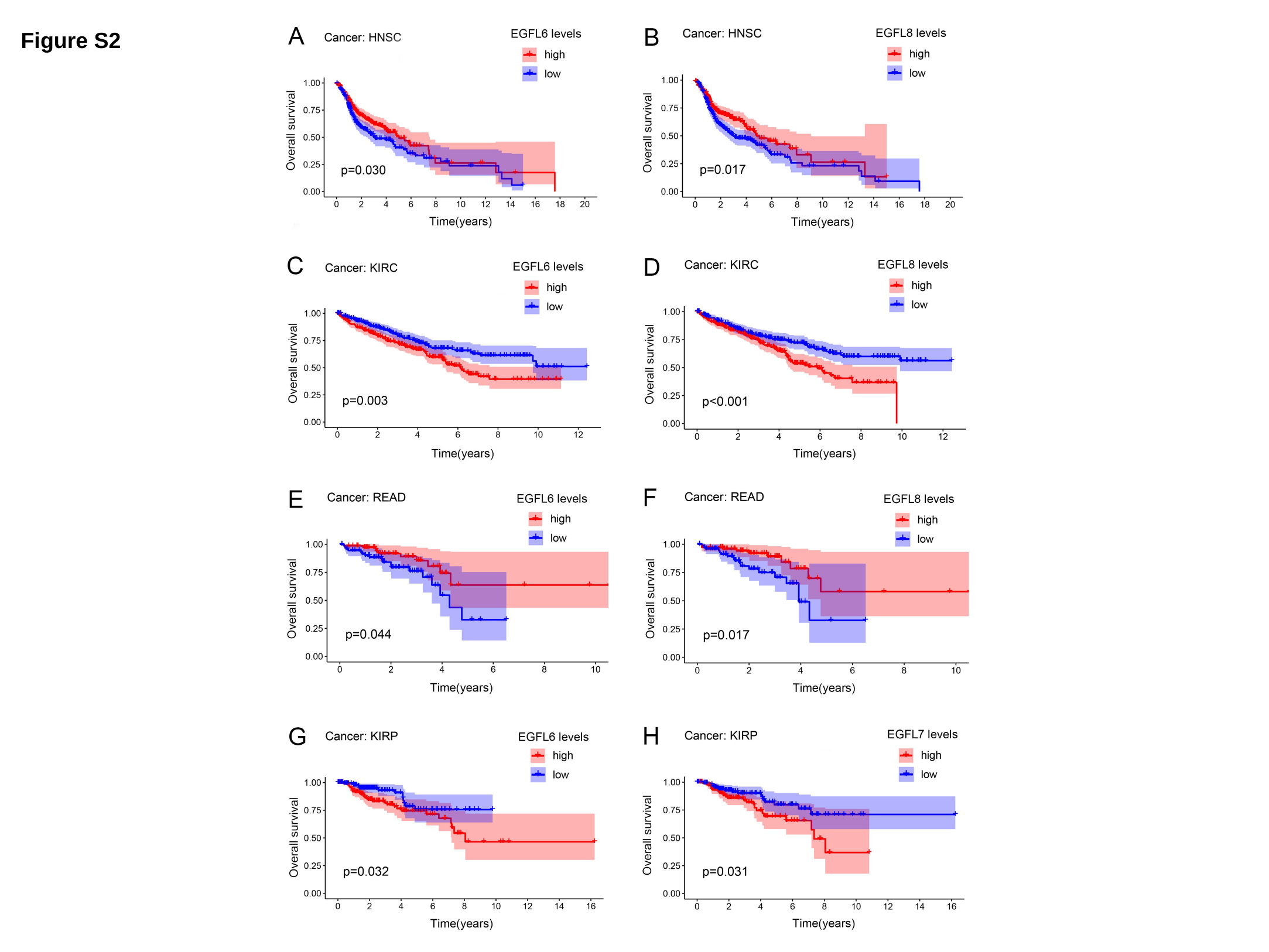

Figure S2

## Slide 3
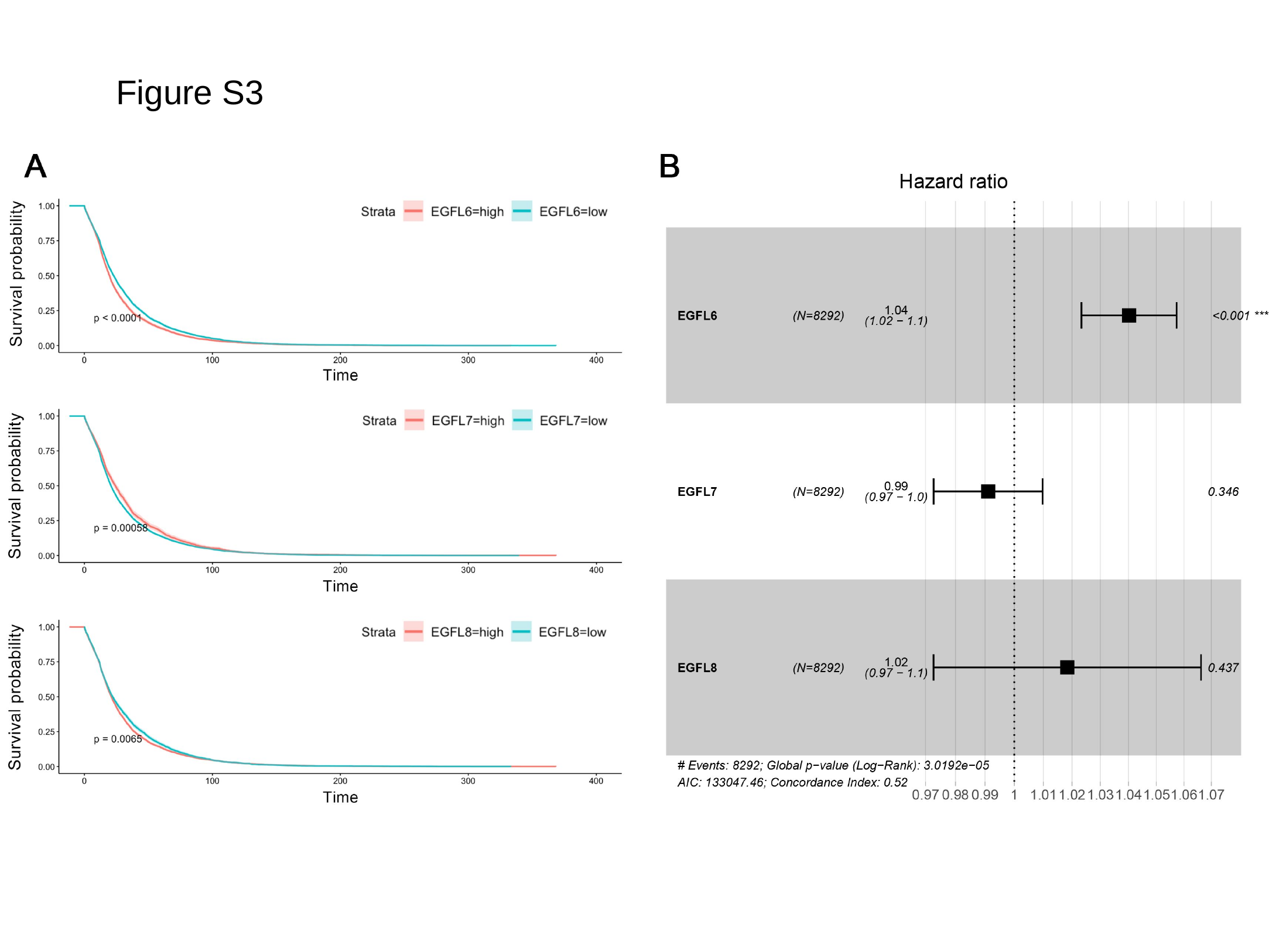

Figure S3

## Slide 4
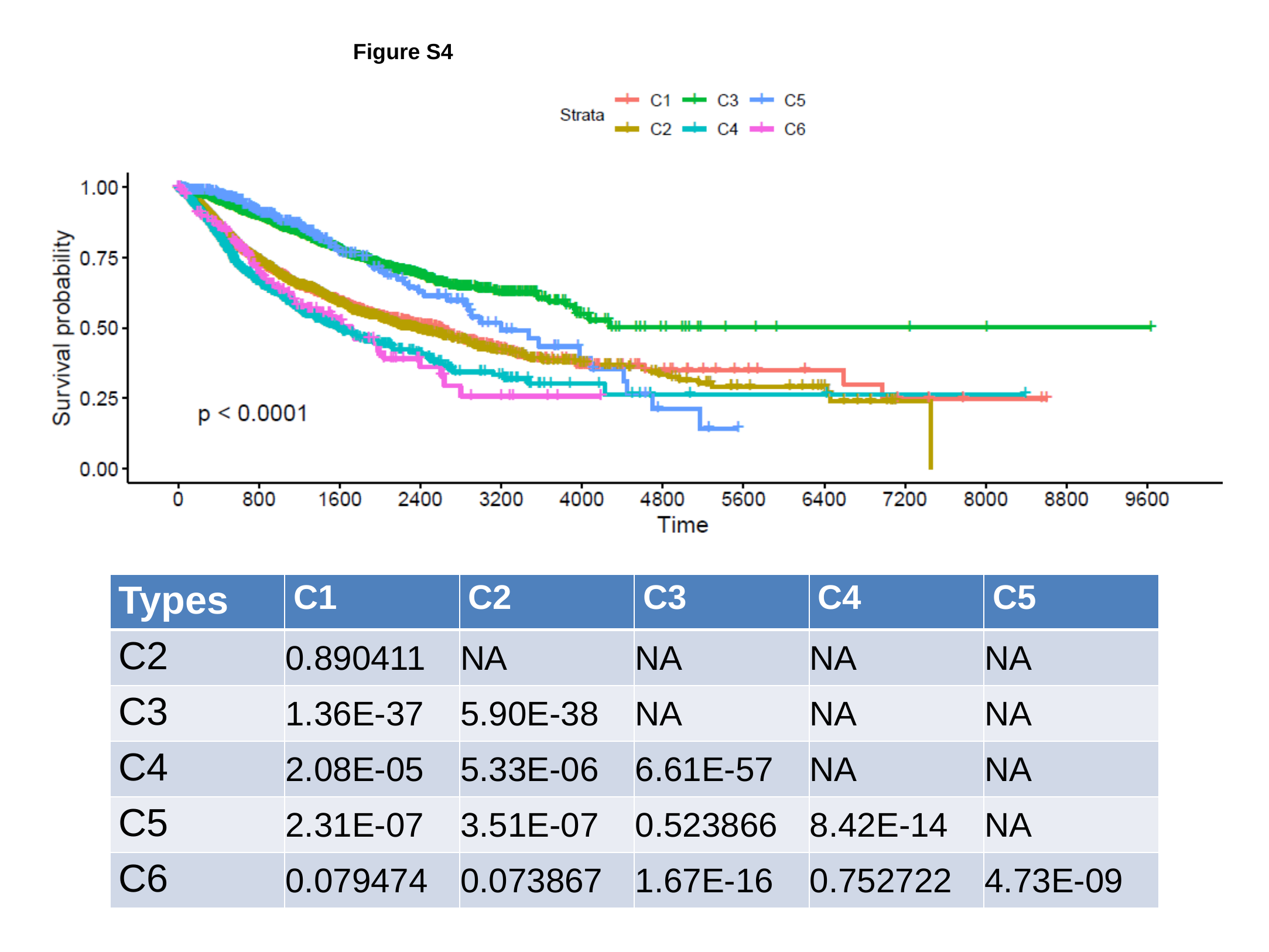

Figure S4
| Types | C1 | C2 | C3 | C4 | C5 |
| --- | --- | --- | --- | --- | --- |
| C2 | 0.890411 | NA | NA | NA | NA |
| C3 | 1.36E-37 | 5.90E-38 | NA | NA | NA |
| C4 | 2.08E-05 | 5.33E-06 | 6.61E-57 | NA | NA |
| C5 | 2.31E-07 | 3.51E-07 | 0.523866 | 8.42E-14 | NA |
| C6 | 0.079474 | 0.073867 | 1.67E-16 | 0.752722 | 4.73E-09 |

## Slide 5
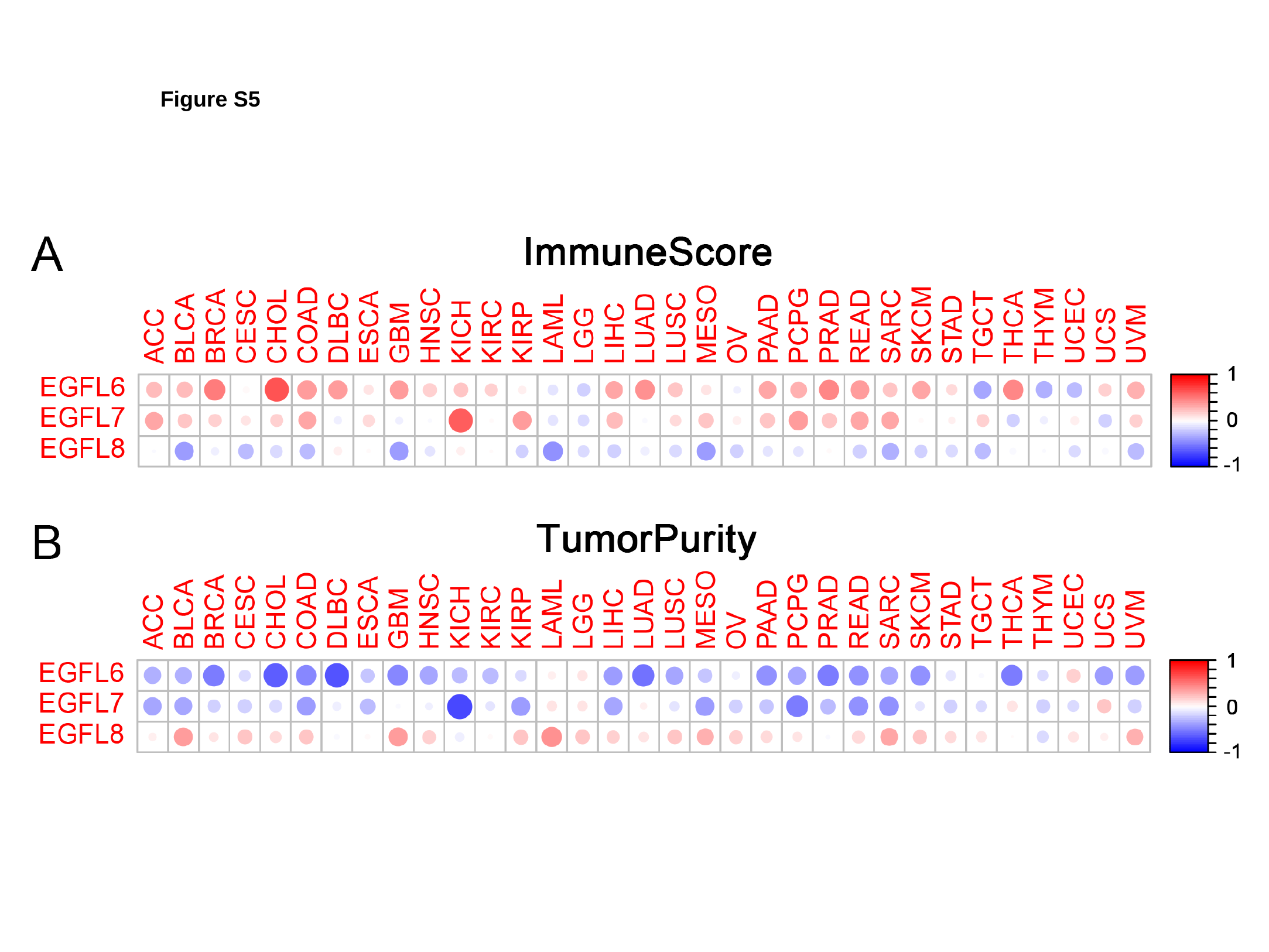

Figure S5

## Slide 6
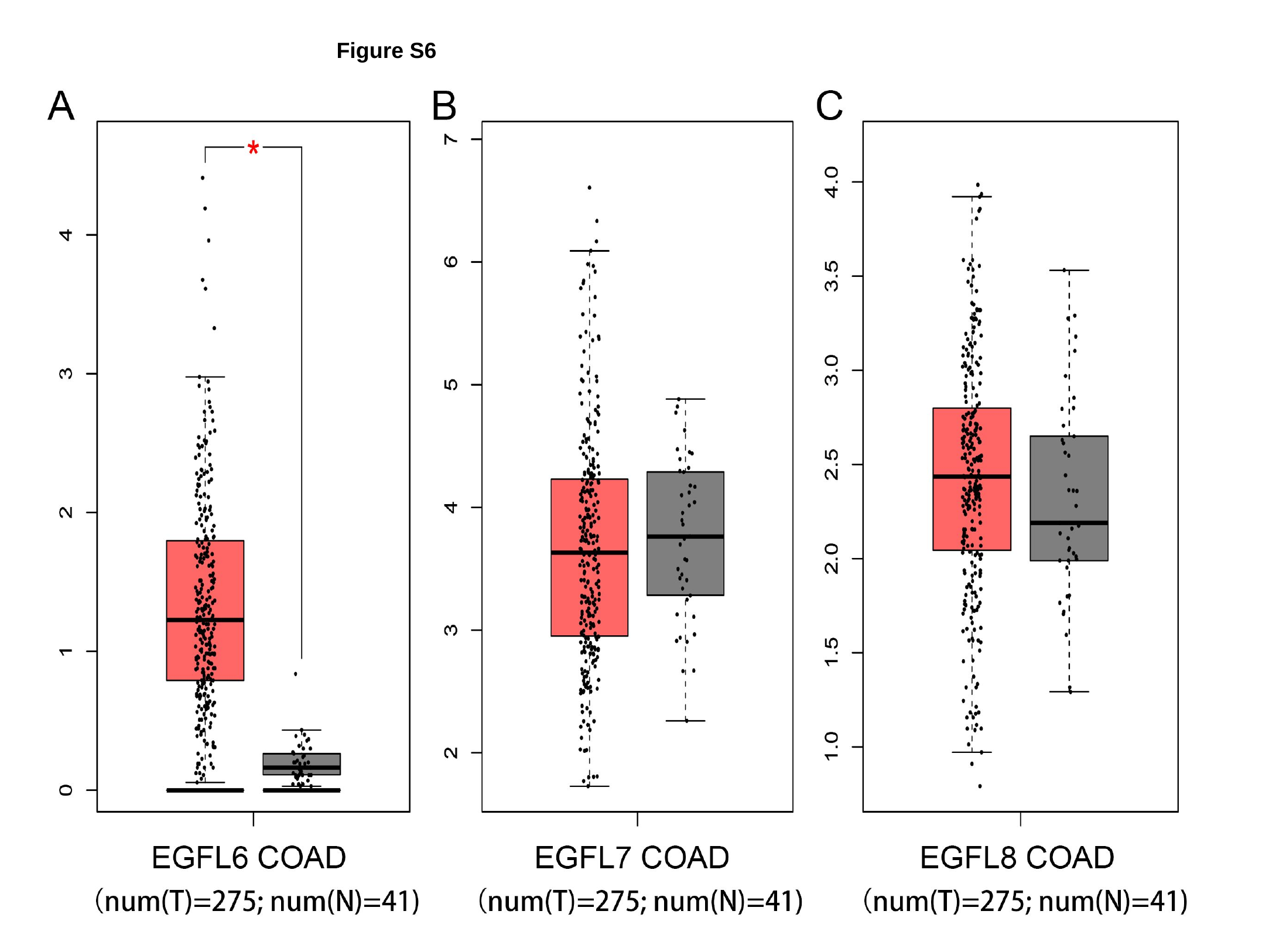

Figure S6
